# THE HEALTHCARE CHALLENGE FOR AN AGING POPULATION: THE ROLE OF TECHNOLOGY

**DOI:** 10.1093/geroni/igz038.124

**Published:** 2019-11-08

**Authors:** Sara J Czaja

**Affiliations:** Weill Cornell Medicine, New York, New York, United States

## Abstract

Although many older adults enjoy relatively good health into their later years, many have one or more chronic conditions, diseases, or disabilities, and need help with disease management activities or activities important to independent living. With the increase in numbers of aging adults there is a concomitant strain on the healthcare system. Models of healthcare are also changing and moving towards a more partnership model where consumers are supposed to assume a more collaborative role in health decision making and a more active role in health management. This presentation will discuss the continuing and increasing role of technology in meeting the healthcare challenges for an aging population. The discussion will focus on technology applications to support the health and well-being of older adults as well as family caregivers. Challenges and barriers that currently limit the full potential of technology to be realized for these populations will also be discussed.

